# IL-17A mediates inflammatory and tissue remodelling events in early human tendinopathy

**DOI:** 10.1038/srep27149

**Published:** 2016-06-06

**Authors:** Neal L. Millar, Moeed Akbar, Abigail L. Campbell, James H. Reilly, Shauna C. Kerr, Michael McLean, Marina Frleta-Gilchrist, Umberto G. Fazzi, William J. Leach, Brian P. Rooney, Lindsay A. N. Crowe, George A. C. Murrell, Iain B. McInnes

**Affiliations:** 1 Institute of Infection, Immunity and Inflammation, College of Medicine, Veterinary and Life Sciences University of Glasgow, Glasgow, Scotland UK; 2 Department of Orthopaedic Surgery, NYU School of Medicine, New York, USA; 3 Department of Orthopaedic Surgery, Queen Elizabeth University Hospital, Glasgow, Scotland, UK; 4 Orthopaedic Research Institute, Department of Orthopaedic Surgery, St George Hospital Campus, University of New South Wales, Australia

## Abstract

Increasingly, inflammatory mediators are considered crucial to the onset and perpetuation of tendinopathy. We sought evidence of interleukin 17A (IL-17A) expression in early human tendinopathy and thereafter, explored mechanisms whereby IL-17A mediated inflammation and tissue remodeling in human tenocytes. Torn supraspinatus tendon (established pathology) and matched intact subscapularis tendon (representing ‘early pathology’) along with control biopsies were collected from patients undergoing shoulder surgery. Markers of inflammation and IL-17A were quantified by RT-PCR and immunohistochemistry. Human tendon cells were derived from hamstring tendon obtained during ACL reconstruction. *In vitro* effects of IL-17A upon tenocytes were measured using RT-PCR, multiplex cytokine assays, apoptotic proteomic profiling, immunohistochemistry and annexin V FACS staining. Increased expression of IL-17A was detected in ‘early tendinopathy’ compared to both matched samples and non-matched control samples (p < 0.01) by RT-PCR and immunostaining. Double immunofluoresence staining revealed IL-17A expression in leukocyte subsets including mast cells, macrophages and T cells. IL-17A treated tenocytes exhibited increased production of proinflammatory cytokines (p < 0.001), altered matrix regulation (p < 0.01) with increased Collagen type III and increased expression of several apoptosis related factors. We propose IL-17A as an inflammatory mediator within the early tendinopathy processes thus providing novel therapeutic approaches in the management of tendon disorders.

Overuse tendon injuries, namely tendinopathies, pose a significant clinical problem, particularly in musculoskeletal and sports related medicine^1^. The intrinsic pathogenetic mechanisms underlying the development of tendinopathies are largely unknown however proinflammatory mediators, such as cytokines, have recently been implicated functionally in several model systems^2^. In particular, the interaction between apoptosis and inflammation is critical to tissue homeostasis and is known to play a key role in diseases such as rheumatoid arthritis^4^, neoplasia^5^, neurodegeneration^6^ and cardiovascular disease^7^.

Cytokines are critical in the response of soft tissues to injury and wound healing and have been shown to be involved in the regulation of matrix turnover in tendinopathy^8^. Endogenous expression of TNFα, IL-1β, IL-6, IL-10, VEGF and TGFβ has been demonstrated in tenocytes^9^,^10^,^11^,^12^. Such expression is functionally implicated *in vivo*: for example, the mechanical properties of healing tendons in IL-6^−/−^ mice were inferior compared with littermate controls. Cytokines also play a key role in oxidative stress-induced cellular apoptosis^13^ which is mediated by the activation of a variety of caspases. IFN-γ or TNF-α increased, whereas TGF-β or IL-10 decreased caspase 8 driven Fas mediated apoptosis in dermal fibroblasts^14^. IL-1β and TNF-α induce mitochondrial DNA damage, and mitochondrial transcription with subsequent induction of apoptosis in human chondrocytes^15^. By this means an inflammatory/apoptotic cascade of molecular events can promote tissue degeneration^16^.

Interleukin-17A, a member of the IL-17 family of cytokines is a pro-inflammatory mediator increasingly implicated in a variety of immune and inflammatory disease states. IL-17A expression is reported in several cell types including Th17 cells^17^, γδT cells^18^ and some non lymphocyte lineages^19^; its receptor is ubiquitously expressed on many cell types, including myeloid cells, epithelial cells and fibroblasts^20^. IL-17A exerts various biological activities that could promote tissue destruction and degeneration during inflammation. In particular, it induces the production, often in a synergistic manner, of cytokines, including IL-1, IL-6, TNF-α, chemokines, inducible NO synthase, and matrix metalloproteinases (MMPs) by fibroblasts, macrophages, and endothelial cells^21^. Experimental models provide evidence for an early inflammatory response in tendinopathy^2^,^2^2,^23^. Recently we have shown a distinct inflammatory infiltrate in early human tendinopathy^24^ and increased levels of proinflammatory cytokines including TNF-α, IL-6 in torn supraspinatus samples^25^. A recent transcriptomic analysis has highlighted increased expression of IL-17F in human tendinopathy biopsies^26^. Based on these observations and the plausible biological profile exhibited by IL-17A, we hypothesized that IL-17A may play a role in tendinopathy.

One of the major limitations of human studies is that tendon biopsies are usually obtained when patients are symptomatic and therefore biopsy material is likely to represent chronic, rather than early phase processes. We previously suggested that matched subscapularis tendon from patients with full thickness rotator cuff tears may be a model of early human tendinopathy based on histological appearances and significantly increased levels of cytokines and apoptotic markers in these tissues^27^. The purpose of this study was to formally assess the expression of IL-17A within this model and thereafter explore the mechanistic activities of IL-17A on inflammation, apoptosis and matrix production in tenocytes *in vitro*.

## Methods

### Human model of tendinopathy

All procedures and protocols were approved by the South East Area Sydney Area Ethics Committee (ACEC No. 99/101) and NHS West of Scotland Ethics Committee (REC 11/S0704/7) with informed consent obtained and carried out in accordance standard operative procedures. Ten supraspinatus tendon samples were collected from patients with rotator cuff tears undergoing shoulder surgery (Table 1). The mean age of the rotator cuff ruptured patients was 54 years (range, 35–70 years) - the mean tear size was 2.5 cm^2^. Samples of the subscapularis tendon were also collected from the same patients. Patients were only included if there was no clinically detectable evidence of subscapularis tendinopathy on a preoperative MRI scan or macroscopic damage to the subscapularis tendon at the time of arthroscopy – by these criteria they represented a truly pre-clinical cohort. In this cohort all patients fulfilled the following criteria: 1) a history of shoulder pain and dysfunction, 2) no previous surgery on the affected shoulder, 3) no radiographic sign of fracture of the shoulder, and 4) no history of rheumatoid or osteoarthritis. An independent control group was obtained comprising 10 samples of subscapularis tendon collected from patients undergoing arthroscopic surgery for shoulder stabilization without rotator cuff tears, no previous shoulder surgery, no radiographic signs of shoulder fracture of history of rheumatoid or osteoarthritis. The absence of rotator cuff tears was confirmed by arthroscopic examination. The mean age of the control group was 35 years (range, 20–41 years).

### Tissue collection and preparation

Arthroscopic repair of the rotator cuff was carried out using the standard three-portal technique while the cross-sectional size of the rotator cuff tear was estimated and recorded as previously described^28^. The subscapularis tendon was biopsied arthroscopically from the superior border of the tendon 1 cm lateral to the glenoid labrum representing mid body tendon structure. The supraspinatus tendon was harvested from within 1.5 cm of the edge of the tear prior to surgical repair. For immunohistochemical staining the tissue samples were immediately fixed in 10% (v/v) formalin for 4 to 6 hours and then embedded in paraffin. Sections were cut to 5 μm thickness using a Leica-LM microtome (Leica Microsystems, Germany) and placed onto Superfrost Ultra Plus glass slides (Gerhard Menzel, Germany). The paraffin was removed from the tissue sections with xylene, rehydrated in graded alcohol and used for histological and immunohistochemical staining per previously established methodologies^29^.

Human tendon derived cells were explanted from hamstring tendon tissue of 5 patients (age 18–30 years) undergoing hamstring tendon ACL reconstruction. Cultures were maintained at 37 °C in a humidified atmosphere of 5% CO_2_ for 28 days. Cells were subcultured and trypsinized at subconfluency, with cells from the 3^rd^ and 4^th^ passage utilised.

### Histology and Immunohistochemistry techniques

Samples were stained with haematoxylin and eosin and toluidine blue for determination of the degree of tendinopathy as assessed by a modified version of the Bonar score^30^ (Grade 4 = marked tendinopathy, Grade 3 = advanced tendinopathy, 2 = moderate degeneration 1 = mild degeneration 0 = normal tendon). This included the presence or absence of oedema and degeneration together with the degree of fibroblast cellularity and chondroid metaplasia. Thereafter, sections were stained with a range of primary monoclonal antibodies directed against the following markers: - IL-17A [using either goat anti-IL-17A polyclonal (R&D Systems, Abingdon, U.K) or mouse anti-IL-17A monoclonal (Cambridge Bioscience,UK), CD68, CD3, CD4, and mast cell tryptase (mast cells) (Vector Labs).

Endogenous peroxidase activity was quenched with 3% (v/v) H_2_O_2_, and nonspecific antibody binding blocked with 2.5% horse serum in TBST buffer for 30 minutes. Antigen retrieval was performed in 0.01 M citrate buffer for 20 minutes in a microwave. Sections were incubated with primary antibody in 2.5% (w/v) horse serum/human serum/TBST at 4 °C overnight. After two washes, slides were incubated with Vector ImmPRESS Reagent kit as per manufactures instructions for 30 minutes. The slides were washed and incubated with Vector ImmPACT DAB chromagen solution for 2 minutes, followed by extensive washing. Finally the sections were counterstained with hematoxylin.

For double immunofluorscencent staining, ten paraffin embedded samples were incubated for 1 h with Abs (mouse anti-CD3 [1.25 μg/ml; Vector Laboratories], mouse anti-CD4 [7.56 μg/ml; Dako UK, Cambridgeshire, U.K.], mouse anti-mast cell tryptase [MCT] [0.43 μg/ml; Dako UK], or mouse anti-CD68 [1 μg/ml; Dako UK]) followed by 30 min incubation with biotinylated Abs (1:200; Vector Laboratories) with subsequent staining with streptavidin DyLight Fluor (1:500; Invitrogen, Paisley, U.K.) for 45 min. A second Avidin/Biotin blocking step (Avidin/Biotin Blocking Kit, Vector Labs) was carried out to prevent cross reactivity with the secondary antibody. Goat anti–IL-17A (5 μg/ml) was added overnight at 4 °C, then incubated with a biotinylated Ab for 30 min, and stained with Avidin FITC (1:500; Vector Laboratories) for 45 min. Slides were mounted with Vectashield containing DAPI (Vector Laboratories) and analyzed on a fluorescent imaging microscope (BX50; Olympus, Essex, U.K.). Images were captured using Apple Open laboratory software. Positive (human tonsil tissue) control specimens were included, in addition to the surgical specimens for each individual antibody staining technique and double immunofluorescence staining. Omission of primary antibody and use of negative control (human osteoarthritis samples) isotypes confirmed the specificity of staining.

We applied a scoring system based on previous methods^31^ to quantify the immunohistochemical staining. Five random high power fields (x400) were evaluated by three independent assessors (NLM, JHR, ALC). In each field the number of positive and negatively stained cells were counted and the percentage of positive cells calculated giving the following semi-quantitative grading; Grade 0 = no staining, Grade 1 = <10% cells stained positive, 2 = 10–20% cells stained positive, Grade 3 = >20% cells positive. Additional sub analysis of IL-17A + cells calculated the actual number of double stained cells were per high power field.

### Apoptosis and Matrix Regulation

Cells were analysed using Annexin V binding, which detects transfer of phosphatidylserine to the outside of the membrane, cells were harvested with trypsin and stained with Annexin V-FITC Apoptosis Detection Kit (BD Pharmagen) for flow cytometer analysis. The production of thirty-five apoptotic related proteins in cell lysates were assessed using a proteome profiler (R&D systems) that contains capture and control antibodies spotted in duplicate on nitrocellulose membranes. The array is incubated with a cocktail of biotinylated detection antibodies.

Total soluble collagen was measured from cell culture supernatants using the Sircol assay kit (Biocolor Ltd, Carrickfergus, Northern Ireland) according to the manufacturer’s protocol. 1 ml of Sircol dye reagent was added to 100 μl test sample and mixed for 30 min at room temperature. The collagen-dye complex was precipitated by centrifugation at 10,000 × *g* for 10 min; and then washed twice with 500 μl of ethanol. The pellet was dissolved in 500 μl of alkali reagent. The absorbance was measured at 540 nm by microplate reader. The calibration curve was set up on the basis of collagen standard provided by the manufacturer. Additionally protein levels for Collagen I and III were quantified using pre coated ELISA plates (USCN Life Science Inc, Europe) with monoclonal antibodies specific for human Collagen I and Collagen III and read at 450 nm by microplate reader after incubation with samples or collagen standards. The concentration of Collagen I and III in the samples was then calculated by comparing the optical density of the sample to the standard curve.

### Signaling experiments

Phosphorylation status of mitogen-activated protein kinases (MAPKs), extracellular signal regulated kinases (ERK1/2), c-Jun N-terminal kinases (JNKs) and p38 isoforms were evaluated using the Human Phospho-MAPK Array (R&D Systems Europe, UK) as per the manufacturer’s instructions. The ERK inhibitor (FR180204) and Atk inhibitor (1L6-Hydroxymethyl-chiro-inositol-2-(R)-2-O-methyl-3-O-octadecyl-*sn*-glycerocarbonate) were purchased from CalbioChem (Merck KGaA, Germany) and used at IC_50_ = 10 μM, a concentration previously determined to offer optimal specific inhibition relative to off target effects.

### RNA Extraction and Quantitative PCR

Tissue from 10 patients was available for RNA analysis and was placed in RNALater (Ambion) at the time of surgery. The cells isolated from *in vitro* experiments along with the available biopsy samples were placed in Trizol prior to mRNA extraction. QIAgen mini columns (Qiagen Ltd, Crawley UK) were used for the RNA clean-up with an incorporated on column DNAse step as per manufacturer’s instructions. cDNA was prepared from RNA samples according to AffinityScript™ (Agilent Technologies, CA, USA) multiple temperature cDNA synthesis kit as per manufactures instructions. SYBR green Real time PCR was performed using SYBR green mastermix (Applied Biosystems, CA, USA). Prior to setting up the SYBR green the cDNA was diluted 1 in 5 using RNase-free water. Each sample was analysed in triplicate. Primers (Integrated DNA Technologies, Belgium) were as follows: GAPDH, 5′-TCG ACA GTC AGC CGC ATC TTC TTT-3′ (F) and 5′-ACC AAA TCC GTT GAC TCC GAC CTT-3′ (R), COL1A, 5′- CAA TGC TGC CCT TTC TGC TCC TTT-3′ (F) and 5′-CAC TTG GGT GTT TGA GCA TTG CCT-3′ (R), COL 3A, 5′- TAT CGA ACA CGC AAG GCT GTG AGA-3′ (F) and 5′-GGC CAA CGT CCA CAC CAA ATT CTT-3′ (R), MMP 13, 5′- AAG GAC CCT GGA GCA CTC ATG TTT-3′ (F) and 5′-TGG CAT CAA GGG ATA AGG AAG GGT-3′ (R), CASPASE 3, 5′-TCA TTA TTC AGG CCT GCC GTG GTA (F) and 5′-TGG ATG AAC CAG GAG CCA TCC TTT-3′, CASPASE 7, 5′-TTC CTC TTC GCC TAT TCC ACG GTT (F) and 5′-ATT CAC CCT GGT GAG GAT CTG CAT-3′ (R), Smac, 5′ GCG CGG ATC CAT GGC GGC TCT GAA GAG TTG (F) and 5′ AGC TCT CTA GAC TCA GGC CCT CAA TCC TCA (R), IL-17A, 5′-AGG CCA TAG TGA AGG CAG GAA TCA-3′ (F) and 5′- ATT CCA AGG TGA GGT GGA TCG GTT-3′ (R).

### Cytokine production

A 25-Plex human cytokine assay evaluated the *in vitro* quantitative determination of 25 separate human cytokines using Luminex technology. Supernatants (n = 5, in triplicate) were removed from cell culture and analyzed for cytokine production.

### Statistical analysis

Results are reported as mean values ± SD. ANOVA followed by Tukey’s test or Student’s t test was applied to *in vitro* studies. Analysis between individual *in vitro* groups was examined by ANOVA followed by the Student-Newman-Keuls test or Student’s t test using Graphpad Prism, version 6.0 (Graphpad Inc, CA). A value of p < 0.05 was considered to be significant.

### Ethical approval information

All procedures and protocols were approved by the South East Area Sydney. Area Ethics Committee (ACEC No. 99/101) and NHS West of Scotland Ethics Committee (REC 11/S0704/7) with informed consent obtained and carried out in accordance standard operative procedures.

### Data Availability

NLM has access to all the data and data are available upon request.

## Results

### Early tendinopathy shows increased IL-17A expression

**Figure 1:**
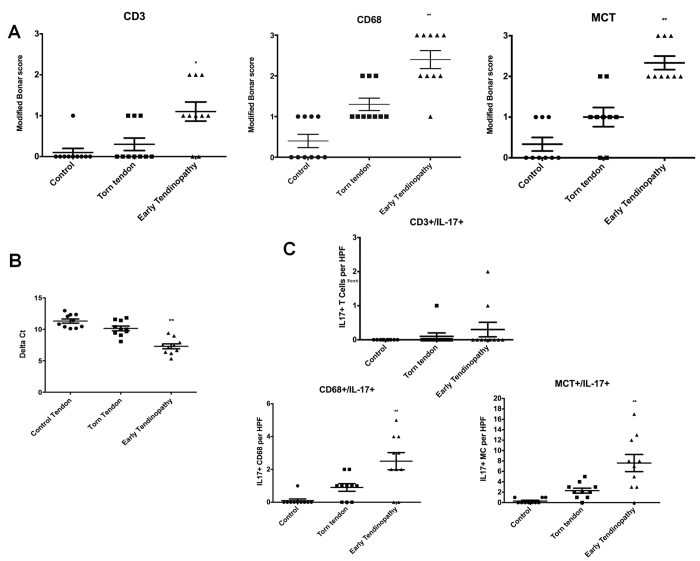
IL-17A expression in human tendinopathy samples. (**A**) Graphs illustrate modified Bonar scoring for samples of human tendon biopsies for expression of CD3, CD68 and MCT markers with mean and SEM shown. n = 10 for control tendon, n = 10 for torn tendon and early tendinopathy. Modified Bonar scoring system depicts mean score per sample based on 10 high power fields. 0 = no staining, 1 = <10%, 2 = 10–20%, 3 = >20% + ve staining of cells per high power field. *p < 0.05, **p < 0.01 (Students *t-*test) (**B**) IL-17A gene expression in tendon samples. Delta Ct values of IL-17A in control (n = 10, intact subscapularis biopsy), torn tendon (torn supraspinatus biopsy) and early tendinopathy (matched intact subscapularis biopsy) human tendon samples (n = 10). Data are mean ± SD relative to house keeping gene18S (mean of duplicate analysis). *p < 0.05, **p < 0.01 (Students *t-*test). (**C**) Quantitative expression of CD3 + /IL-17+, CD68 + /IL17+ and MCT/IL-17+ depicts mean cells per sample based on 10 high power fields. n = 10 for control tendon, n = 10 for torn tendon and early tendinopathy *p < 0.05, **p < 0.01 versus control tissue, (Students *t-*test).

**Figure 2:**
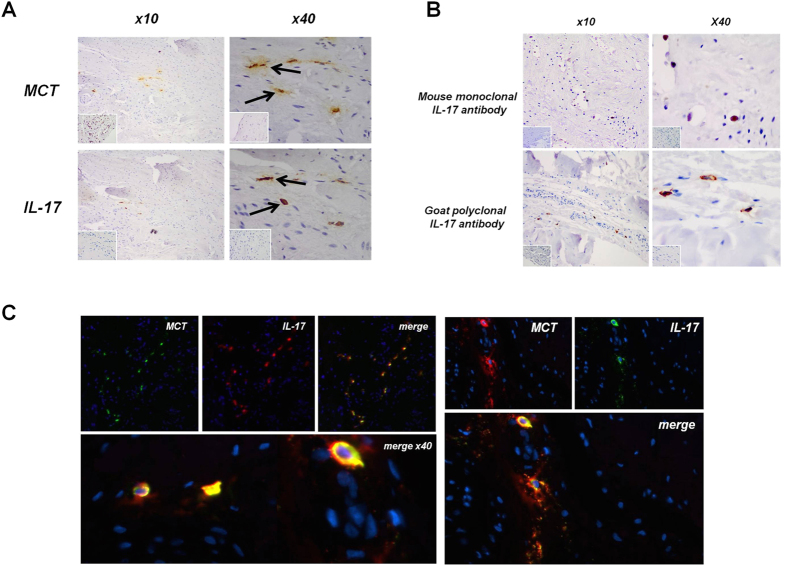
IL-17A localisation in tendon samples. (**A**) Early human tendinopathy sample (matched subscapularis tendon biopsy) stained for (**A**) Mast cell tryptase (MCT) and IL-17A, Isotype IgG in bottom left corner using goat polyclonal IL-17A antibody. (**B**) Early tendinopathy sample stained for IL-17A using mouse monoclonal antibody and goat polyclonal antibody. Isotype IgG in bottom left corner. (**C**) Double imunofluorescence staining using goat polyclonal IL-17A antibody showing IL-17A, Mast Cell Tryptase and double staining co-localization in early tendinopathy sample. (x100).

Staining for cell markers of macrophages, mast cells and T cells was significantly greater in subscapularis compared to both matched torn supraspinatus biopsies and control tissues (Fig. 1A), confirming that our early tendon lesions contained an inflammatory infiltrate. Subscapularis tendon biopsies exhibited significantly (p < 0.01) greater IL-17A mRNA levels than did biopsies from torn supraspinatus or control tendon (Fig. 1B). We also observed increased tissue staining for IL-17A. To characterise these IL-17A^+^ cells in human tendinopathy, we performed colocalisation studies and calculated the proportion of IL-17A^+^ cells contained in each cellular subset. We first investigated T cell markers, namely, CD3, CD4, that colocalised with IL-17A (Fig. 1C). Although some CD3^+^ IL-17A^+^ cells were identified, the majority of IL-17A^+^ cells were CD3-negative. Approximately 1% of IL-17A expressing cells expressed CD3 by IHC. CD4 cells were limited in number throughout the samples and we identified no co-expression of IL-17A, presumably reflecting this low sample size. Up to 20% of IL-17A^+^ cells were CD68^+^ (Fig. 1C and Supplementary Fig. 1A). The majority of IL-17A^+^ cells double-stained with MCT, suggesting that mast cells represent a lineage in which IL-17A protein expression is enriched in human tendinopathy (Figs 1C and 2A,C). As a further control, we used both goat polyclonal and mouse monoclonal anti-human IL-17A antibodies and obtained essentially similar staining patterns in tissue (Fig. 2B).

The inflammatory cell infiltrate correlated inversely (r = 0.4, p < 0.01) to rotator cuff tear size in the torn supraspinatus biopsies with larger tears showing significantly fewer inflammatory cell types. No significant associations were noted between IL-17A expression and the age of patients, duration of symptoms or number of prior steroid injections. All torn supraspinatus samples showed Grade 4 changes consistent with marked degeneration, mucoid change and frank chondroid metaplasia. Matched subscapularis tendon showed Grade 2–3 changes indicative of moderate-advanced tendinopathy. All control samples were classified as Grade 1 consistent with normal fibrotendinous tissue with large distinct collagen fibrils. There were no significant correlations between Bonar score and the mean duration of symptoms or age of the patient cohort.

### IL-17A alters tenocyte collagen synthesis

**Figure 3:**
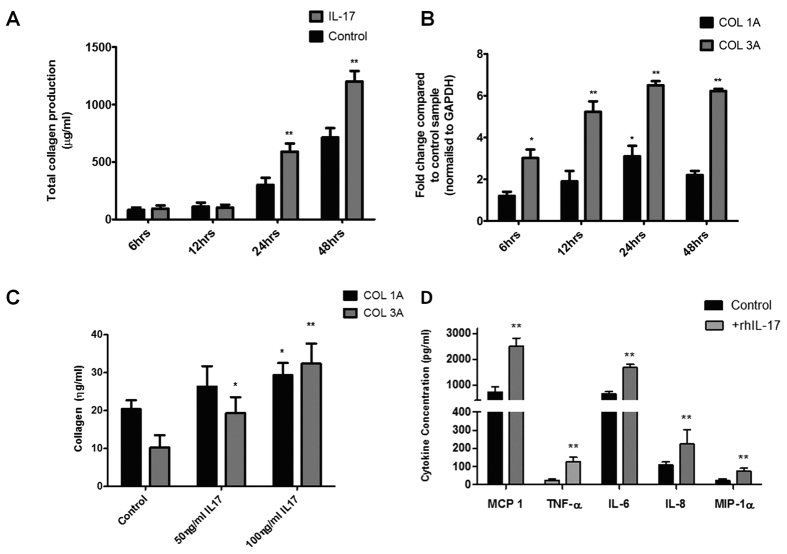
The effect of IL-17A on collagen matrix production. (**A**) Total collagen production assessed using Sircol assay. Data represents mean collagen production (μg/ml) ± SD in control and rhIL-17A(100 ng) treated normal hamstring tenocyte cultures over a 48 hour time course. *p < 0.05, **p < 0.01 compared to control samples.(Students *t*-test) (**B**) The levels of mRNA for Collagen type IA and Collagen type IIIA were determined by real time PCR over a 48 hour time (100 ng rhIL-17A). Data shown as the mean ± SD of triplicate samples and represent experiments on five individual normal hamstring tendon patient explant samples utilising GAPDH housekeeping versus control. *p < 0.05, **p < 0.01 (Students *t*-test) (**C**) Collagen I and III protein expression 24 hours post incubation in control versus 50/100 ng rhIL-17A conditions. Data are shown as the mean ± SD of triplicate samples and are in turn, representative of experiments performed on three individual normal hamstring tendon patient explants. *p < 0.05, **p < 0.01 compared to control samples (Students *t*-test). (**D**) Cultured normal hamstring tenocytes were incubated with recombinant IL-17A (100 ng) over 24 hours. Data shown represent levels of MCP-1, TNF-α, IL-8, IL-6 and MIP-1α in supernatants removed from culture at 24 hrs analysed using Luminex. Data shown are the mean ± SD of triplicate samples and are representative of five individual experiments *p < 0.05, **p < 0.01 (ANOVA).

Since type III collagen expression is a dominant feature of tendinopathy, as compared with type I collagen, we next considered whether IL-17A might alter differential collagen synthesis by tenocytes utilising a previously characterized *in vitro* system^31^. rhIL-17A (using dose/time points optimization derived in preliminary experiments) significantly elevated total collagen production by tenocytes (Fig. 3A) at 24 hours (mean 300 μg/ml ± 62 control; 590 μg ± 71 IL-17A) and 48 hours (712 μg ± 82 control; 1200 μg ± 92 IL-17A). Collagen 1A mRNA levels were unchanged at 6, 12 and 48 hours whereas in contrast, Collagen 3A mRNA was significantly up-regulated at 24 & 48 hours by rhIL-17A compared to controls (Fig. 3B). This was confirmed at the protein level showing a significant increase in collagen III production at 24 hours (mean 10.2 ηg/ml ± 3 control; 32.4 ηg ± 5 100 ηg rhIL-17) and 48 hours (mean 16.4 ηg ± 4 control; 45.5 ηg ± 7 100 ηg rhIL-17) post treatment with rhIL-17A (Fig. 3C).

### IL-17A promotes cytokine production in tenocytes

We next explored the extent to which IL-17A could regulate the local cytokine milieu via modulating tenocyte behaviour. IL-17RA expression was confirmed in both patient biopsy samples and cultured human tenocytes (Supplementary Figure 1B). rhIL-17A (using dose/time points optimization derived in preliminary experiments) significantly elevated production of TNF-α, MIP1α, IL-6, IL-8 and MCP-1 (Fig. 3D). In contrast, we found no production over time of a range of other cytokines including IL-4, IL-5, IL-10, IL-12, IL-13, and IL-15, consistent with our expectations of the known cellular sources of such cytokine production.

### IL-17A induces apoptosis in tenocytes *in vitro*

**Figure 4:**
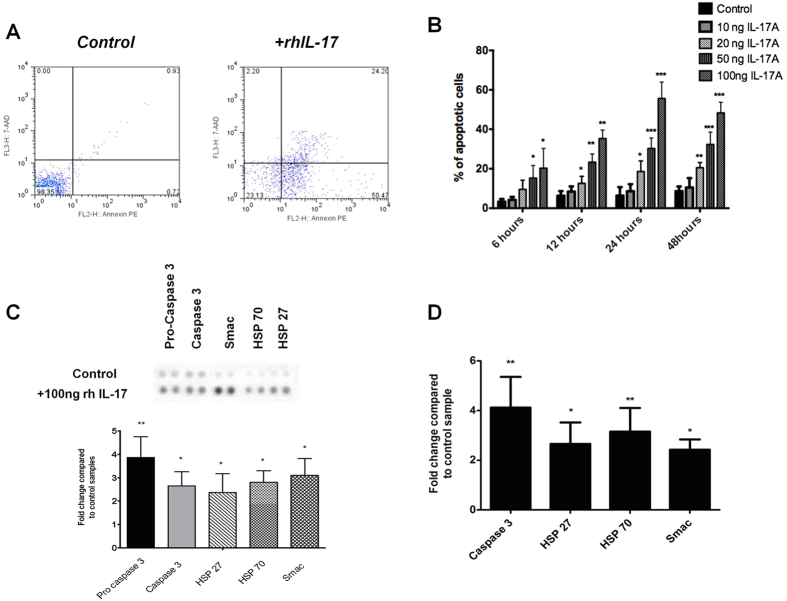
Apoptosis and IL-17A. Apoptosis was evaluated after 24 h of (**A**) control conditions (no cytokines) and rhIL-17A stimulation (100 ng). Flow cytometry profile represents Annexin V-FITC staining in x axis and 7-AAD in y axis. (**B**) Time course showing percentage of apoptotic cells over a 48 hour time course under control (no cytokines) and 10 ng/20 ng/50 ng/100 ng rh IL-17A conditions. Data shown as the mean ± SD of triplicate samples and are in turn, representative of experiments performed on three normal hamstring tendon patient explants. *p < 0.05, **p < 0.01 compared to control samples (ANOVA). (**C**) Whole cell lysates from control and rhIL-17 (100 ng) conditions (24 hours incubation) were harvested and apoptotic markers evaluated using an apoptotic proteome profiler. The fold change of apoptotic proteins was determined by densitometry and normalised to the control sample on the array at 24 hours. *p < 0.05, **p < 0.01 compared to control samples (Students *t*-test). (**D**) The levels of mRNA for the highlighted apoptotic markers were determined by real time PCR under the same conditions (100 ng rh IL-17A at 24 hours). Data are shown as the mean fold change ± SD of duplicate samples and are representative of experiments using five individual donors of normal hamstring tendon explant tissue *p < 0.05, **p < 0.01 compared to control samples (Students *t*-test).

Based on previous observations of dysregulated apoptosis in tendinopathy^31^ and that of IL-17A induced apoptosis in other musculoskeletal disorders we examined the effect of rhIL-17A in our model system *in vitro*. Addition of 100 ng of rhIL-17A to human tenocyte cultures significantly increased the number of cells undergoing apoptosis assessed by Annenix V staining at 24 hours (control mean 5 ± 2%, IL-17A mean 48 ± 10%) and 48 hours (control 8 ± 4%, IL-17A 50 ± 15%) (Fig. 4A,B). Based on our previous observations concerning cell death in early tendinopathy, we utilised limited proteomic profiling to assess which apoptotic mediators may be regulated by IL-17A. This revealed over expression of pro and cleaved caspase 3, heat shock proteins (HSP) 27, 70 and second mitochondria-derived activator of caspases (Smac) (p < 0.01) after 24 hours treatment with 100 ng rhIL-17A. (Fig. 4C and Supplementary Fig. 1C). This was confirmed at the mRNA level by quantitative PCR that revealed elevated expression of caspase 3, HSP 27, HSP 70 and Smac (p < 0.05) in tenocytes (Fig. 4D).

### MAP Kinases mediate some of the effects of IL-17A in tenocytes

**Figure 5:**
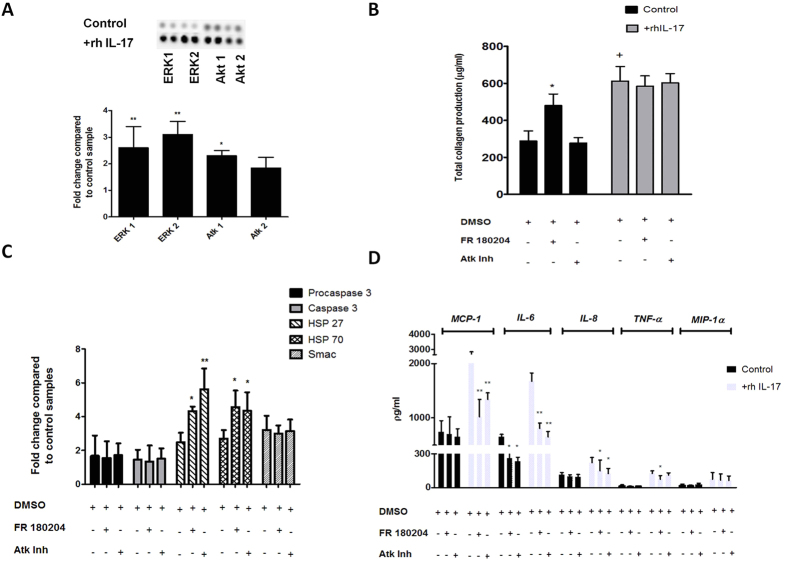
IL-17A induced phosphorylation of MAPK in cultured tenocytes and matrix response to ERK and Atk inhibition. (**A**) Whole-cell lysates from tenocytes were examined for the expression of phosphorylated ERK1, ERK2, Atk1 and Atk2 24 hours after exposure to 100 ng rhIL-17A The fold change of MAPK’s was determined by densitometry and normalised to the control sample on the array. Data are shown as the mean fold change ± SD of duplicate samples and are representative of experiments using three individual donors of normal hamstring tendon explant tissue. *p < 0.05, **p < 0.01 compared to control samples (ANOVA). (**B**) Cells were preincubated for 24 h with specific inhibitors for ERK (FR 180204)(10 μM), or Atk(5 μM) which also were included in the assay media. Total collagen production was assessed using the Sircol assay *p < 0.05, **p < 0.01 (Students *t*-test). (**C**) The levels of apoptotic proteins induced following incubation with ERK and Atk inhibitors were determined (apoptotic proteome profiler) by densitometry and normalised to the relevant control sample on the array. Data are shown as the mean fold change ± SD of duplicate samples and are representative of experiments using five individual donors of normal hamstring tendon explant tissue *p < 0.05, **p < 0.01 (ANOVA). (**D**) Cells were preincubated for 24 h with 100 ng recombinant IL-17A and specific inhibitors for ERK (FR 180204) and Atk, which also were included assay media. Data shows levels of MCP-1, IL-8, IL-6, TNF-α and MIP-1α in supernatants removed from culture analysed using Luminex. Data shown are the mean ± SD of triplicate samples and are representative of five normal hamstring tendon explant experiments *p < 0.05, **p < 0.01 (Students *t*-test).

We next addressed potential signal pathways mediating these effects based on previous reports of MAPK signaling by IL-17A^32^. Tenocytes cultured with rhIL-17A exhibited increased phosphorylation of ERK 1, ERK 2 and Atk 1 & 2 (Fig. 5A and supplementary Figure 1D). To explore the functional consequences of these observations, we employed ERK and Atk inhibitors in our tenocyte cultures. We observed significant reduction in production of MCP-1, IL-6, and IL-8 with both ERK and Atk inhibition, while TNF-α production was reduced by ERK inhibition and not Atk inhibition. No change was noted in MIP-1α production by either inhibitor (Fig. 5D). Interestingly, we observed no significant change in total collagen production, collagen I and III mRNA or protein levels in IL-17A treated tenocytes with ERK or Atk inhibition suggesting that other pathways may regulate those events (Fig. 5B). Additionally ERK and Atk inhibition resulted in a significant increase in HSP 27 and 70 gene expression with no associated changes in caspase 3, or Smac (Fig. 5D) suggesting that ERK/Atk pathways may be involved in modulating the specific pathways implicated in cell death.

## Discussion

Our study provides evidence that IL-17A could operate as a cytokine modulator of early tendinopathy. Herein we demonstrate that IL-17A is present in early tendinopathy biopsies and thereafter in mechanistic studies demonstrate that IL-17 likely contributes to regulation of inflammatory and apoptotic pathways in tendon cells, associated with a change in collagen matrix synthesis.

**Figure 6:**
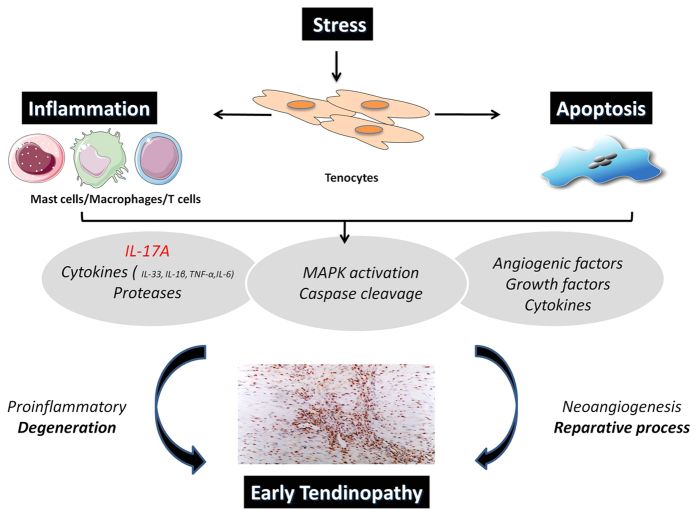
The role of IL-17A in inflammatory mechanisms in tendinopathy. Schematic diagram illustrating the manner in which early tendinopathy may arise due to IL-17A induction. An increase stress that a tendon cell experiences results in the release of various inflammatory mediators such as IL-17A that interact to drive the tendon matrix toward a degenerative of reparative process.

Experimental models provide good evidence of an early inflammatory response in tendinopathy. A running rodent model of tendinopathy is associated with upregulation of key inflammatory modulators including the 5-lipoxygenase activating protein (FLAP) and cyclo-oxygenase^2^ at early and intermediate time points. In rabbit and equine models excessive mechanical load promotes acute inflammatory cell infiltration^22^,^2^3. Our data demonstrate in human tendinopathy that IL-17A is upregulated and thereafter capable of inducing other inflammatory cytokines that may ultimately disturb the balance between reparative and degenerative processes in the extracellular matrix. In particular increased levels of IL-6 and IL-8, which have previously been shown to effect matrix metalloproteinases production in fibroblasts^33^, will likely mediate downstream effects on tenocyte collagen matrix production. Thus the IL-17 cytokine family may provide a molecular link between the initiation and perpetuation of the inflammatory cascade in tendon biology (Fig. 6C). This may not be restricted to IL-17A - increased IL-17F expression from human tendinopathic biopsies is also reported^26^.

We and others have highlighted the key role of infiltrating leukocytes in human tendon disease^24^,^3^4,^35^. The exact cellular source of IL-17A remains uncertain in the context of human inflammatory lesions as compared to disease models^36^,^3^7. Many human biopsy studies suggest IL-17A expression in varied cellular lineages. Whereas lymphoid lineages, particularly T cells and innate lymphocytes are clearly primary IL-17A sources, it is possible that mast cells^37^, macrophages^38^ or neutrophils^39^ can also contribute to the local cytokine pool, either by synthesis, or by receptor mediated uptake and subsequent release. Regardless of the precise cellular synthetic source, our data add growing credence to the notion that immune cells are an early source of IL-17A in response to stress and injury^19^. Investigations in renal fibrosis reveal a role for IL-17RA as a novel modulator of monocyte phenotype and polarisation towards tissue macrophages and thus potentially tissue remodelling^40^. Strategies to interfere with IL-17A signaling and the ability of ERK/Atk inhibitors to reduce IL-17A associated effector function therefore may provide novel therapeutics in human tendinopathy.

Approximately 95% of collagen in normal tendon is type I; type III is present in small amounts. Biopsies from normal and ruptured Achilles tendon demonstrate that ruptured tendons contained reduced quantities of type I collagen as a well as a significant proportion of type III collagen^41^. The presence of type III collagen accounts for the decreased resistance of tendon to tensile forces and subsequent rupture^42^. Here we show that IL-17A not only increased total collagen production but apparently shifted production towards collagen III compared to type I. By this route, IL-17A may cause detrimental phenotypical changes in the tendon extracellular matrix. This idea is supported by recent investigations in scleroderma patients showing decreased collagen I expression in IL-17A treated fibroblasts^43^. Inhibition of ERK/Atk signaling had no effect on IL-17 induced matrix production suggesting an alternative pathway is implicated in this facet of IL-17A effector function. Further investigation is required to understand the signaling biology of IL-17A on collagen synthesis and thus fully appreciate optimal translational targets.

Excessive apoptosis has been postulated as a primary cause of tendinopathies^44^. The apoptotic cells in the rotator cuff have been identified as fibroblasts or fibroblast ‘like’ which are, in turn, key regulators of tendon extracellular matrix^16^, however the regulation of apoptosis in tendinopathy is poorly understood. Several factors, including mechanical overuse^45^, hypoxia^46^ and oxidative stress^47^ are thought to contribute to pathogenesis. We have previously shown increased expression of pro-apoptotic genes in a rodent overuse models and in torn human supraspinatus tendons compared to controls^27^. Our study now reveals that addition of IL-17A to normal tenocyte cultures causes overexpression of caspases molecules known for their ‘death’ role in apoptosis^48^ at both the protein and mRNA level supporting the concept of IL-17A as a further regulator of apoptosis. This reflects *in vitro* findings in osteoarthritis fibroblasts where addition of rhIL-17A promotes apoptosis^49^. However we note that in RA synviocytes rhIL-17A mediates pro survival mechanisms via Bcl-2^50^. Thus further mechanistic work in diseased tenocytes may be required to determine the hierarchical and discrete kinetic roles of IL-17A mediated apoptosis in early versus late tendinopathy.

We also reveal significant changes in several additional regulators of the mitochondrial pathway of apoptosis. HSP 27 indirectly interferes with cell death due to its ability to modulate intracellular glutathione, a parameter that is also regulated by exercise, while HSP 70 interacts with Apaf-1 thereby preventing its interaction with the caspases preventing apoptosis^49^,^51^. Smac binds to inhibitor of apoptosis proteins (IAPs) and deactivates them, preventing the IAPs from suppressing caspase activity^52^. We have previously suggested heat shock proteins as modulators of apoptosis in early tendinopathy^27^ and our mechanistic studies are commensurate with this notion.

There are limitations inherent in our study. Age-related changes within the tendon samples could contribute to the degenerative picture and inflammatory cell expression seen in the matched subscapularis tendons. However the lack of degenerative change on MRI and arthroscopic examinations suggests that the differences are truly at the cellular level as suggested by our work. Subscapularis tendon is functionally and organizationally distinct from supraspinatus and thus responds to mechanical loading in a different manner that may alter its cellular profile. Control samples from subscapularis undergoing stabilisation may not be truly ‘normal’ controls but are currently the best available control tendon sample and this is reflected by a Bonar score of 1. It is reassuring however that we found the same inflammatory cell subtypes in matched subscapularis tissue indicating that the inflammatory response may be uniform within joints subjected to tendon degeneration. In addition having subscapularis samples from the same patient eliminates bias that may result from variation between individuals and has been previously shown to be useful method in sampling of tissues. We point out also that while our human tissue biopsies show presence of IL-17A at mRNA and protein levels, the majority of our mechanistic work utilises an early disease *in-vitro* culture system and that further mechanistic investigation in an *in-vivo* tendon model and late disease *in vitro* cultures would more likely address the hierarchal role of IL-17A in tendon remodelling.

## Conclusion

On the basis of these results we propose IL-17A as an inflammatory regulator in tendon remodeling - better understanding of the pathological cascade that it induces should lead to the development of cell targeted treatment modalities for early human tendinopathy.

## Additional Information

**How to cite this article**: Millar, N. L. *et al.* IL-17A mediates inflammatory and tissue remodelling events in early human tendinopathy. *Sci. Rep.* **6**, 27149; doi: 10.1038/srep27149 (2016).

## Supplementary Material

Supplementary Information

## Figures and Tables

**Table 1:** Patient demographics and rotator cuff tear size.

Tear Size	Control	Small (<1 cm^2^)	Medium (>1–3 cm^2^)	Large (>3–5 cm^2^)	Massive (>5 cm^2^)
Number of cases	10	3	3	2	2
Mean age in years(range)	32 (17–38)	54 (38–62)	55 (46–62)	52 (42–58)	60 (45–70)
Mean duration of symptoms in months (range)	6.0 (2–13)	8.8 (2–20)	6.3 (2–12)	9.8 (5–24)	7.3 (2–20)
Mean number of steroid injections	0	1.6	1.8	1.2	2.1
