# Supplementary material for: IL-17A mediates inflammatory and tissue remodelling events in early human tendinopathy
Source: Sci Rep. 2016 Jun 6;6:27149. doi: 10.1038/srep27149 (PMC4893609; doi:10.1038/srep27149)
Supplement: Supplementary Information [file srep27149-s1.pdf]

SREP-15-35601A

IL-17A mediates inflammatory and tissue remodelling events in early human tendinopathy

Neal L Millar[1], Moeed Akbar[1], Abigail L Campbell[2], James H Reilly[1], Shauna C Kerr[1], Michael McLean[1], Marina Frleta-Gilchrist[1], Umberto G Fazzi[3], William J Leach[3], Brian P Rooney[3], Lindsay AN Crowe[1], George A C Murrell[4] and Iain B McInnes[1]

Supplementary Information

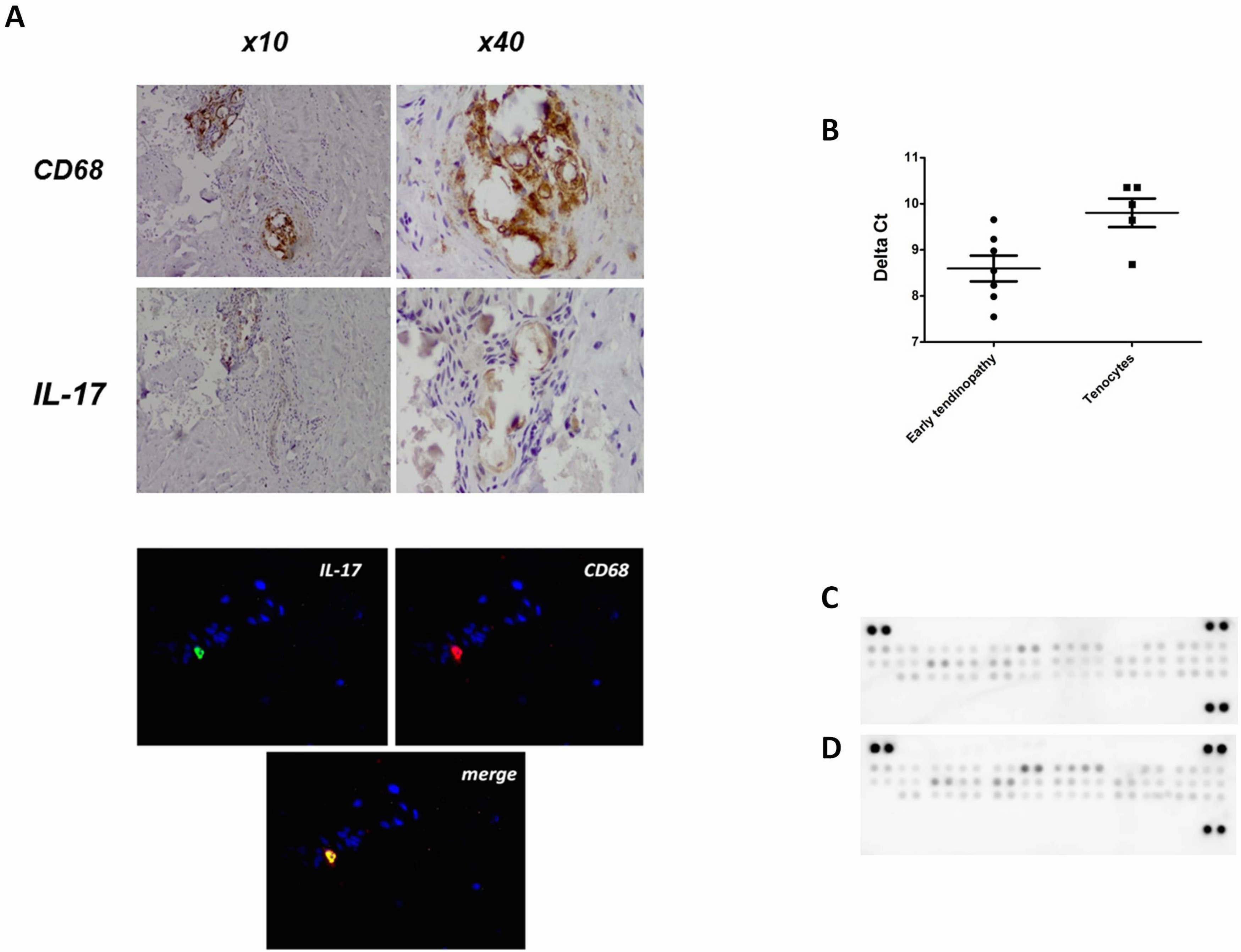
A
x10
x40
CD68
IL-17
IL-17
CD68
merge
B
Delta Ct
11
10
9
8
7
Early tendinopathy
Tenocytes
C
D

Supplementary Figure 1

(A) The levels of mRNA (Delta Ct) for IL-17R were determined by real time PCR. Data shown as the mean ± SD of seven individual early tendinopathy (matched subscapularis) patient tissue samples and five individual control (control hamstring tendon) tenocyte samples with each data point representing the experiment carried out in duplicate. *p<0.05, **p<0.01 (Students *t*-test).
(B) Immunofluorescence staining of purified human mast cells with anti CD4, CD3 and CD20 antibodies to confirm mast cell phenotype. Immunofluorescence staining of purified human mast cells with mast cell tryptase (MCT) and goat polyclonal anti-human IL-17A and mouse monoclonal anti-human IL-17A antibodies
(C-D) Human Phospho-MAPK Array images of experiments depicted in Figures 3C and 5A showing loading controls in upper outer left/right and lower right positions on the array. (E) Immunohistochemistry and double immunofluorescence for IL-17A and CD68 Large areas of CD68 positive areas with single light microscopy suggesting double positive cells. Confirmed on immunofluorescence. Sections were counterstained with DAPI (blue). Bottom row show merged staining. Images are shown at 40x magnification.
